# Outcomes of patients admitted with acute, severe ulcerative colitis on biologic therapy: a retrospective analysis from a tertiary referral hospital

**DOI:** 10.1093/jcag/gwae017

**Published:** 2024-05-31

**Authors:** Nasruddin Sabrie, Manisha Jogendran, Rohit Jogendran, Laura E Targownik

**Affiliations:** Department of Medicine, University of Toronto, Toronto, ON M5S 3HS, Canada; Department of Medicine, Queen’s University, Kingston, ON K7L 3N6, Canada; Department of Medicine, University of Toronto, Toronto, ON M5S 3HS, Canada; Division of Gastroenterology, Mount Sinai Hospital, Toronto, ON M5S 3H2, Canada

**Keywords:** ulcerative colitis, biologic therapy, readmission

## Abstract

**Background:**

In steroid-refractory acute, severe, ulcerative colitis (ASUC), salvage medical therapy with infliximab is recommended to reduce the risk of colectomy. However, the evidence supporting this practice is based on cohorts naïve to biologics. Consequently, the management of patients on biologic or small molecule therapy (BST) with ASUC is not well defined.

**Methods:**

We conducted a retrospective chart review of patients admitted with ASUC to Mount Sinai Hospital (MSH) in Toronto, Ontario from January 2018 until January 2022. Included subjects were considered to be on BST if they had received a dose of these agents within 56 days prior to admission. Our outcomes of interest included the mean difference in hospital length of stay (HLOS), rates of surgical consultation, rates of inpatient colectomies, and 90-day readmission rates between the 2 groups.

**Results:**

Of the 185 admissions for ASUC, 76 were on BST prior to admission and 109 were not. Baseline characteristics were similar between the 2 groups. There were no significant differences in hospital length of stay (7.46 days vs 7.45 days *P* = .52) or in-hospital colectomy rates between the 2 groups. Patients on BST had higher rates of surgical consultation (36.8% vs 8.3% *P* < .01) and 90-day readmission rates (26.3% vs 13.8% *P* = .03).

**Conclusions:**

We did not identify significant differences in the majority of our outcomes between the 2 groups. However, patients on BST were more likely to receive a surgical consultation during their admission and had higher rates of readmission at 90 days. Further studies evaluating the underlying factors that contribute to readmission in patients on BST in hospitals are needed.

**Key message**

**What is already known here?**

a. Rescue therapy for steroid refractory ASUC is supported by observational and interventional studies that excluded patients with current and often prior biologic therapy exposure.

**What is new here?**

b. We demonstrate that patients on BST have similar clinical courses in hospital, compared to patients not on BST. However, they are more likely to receive surgical consultation in hospital and are much more likely to be readmitted within 90 days.

**How can this study help patient care?**

c. Our findings suggest that patients on BST should be more closely monitored post discharge for early signs of severe relapse.

## Introduction

Ulcerative colitis (UC) is a chronic, inflammatory bowel disease (IBD), characterized by continuous inflammation of the colon.^1^ Acute, severe, ulcerative colitis (ASUC), as defined by the Truelove and Witts criteria, is a manifestation of UC that is associated with significant morbidity.^2^ In patients with UC, the lifetime risk of severe colitis is estimated to be 25% with approximately 10%–15% of patients presenting with ASUC at diagnosis.^2,3^ Despite our medical advances in treating ASUC, failure of medical management is a frequently encountered scenario with colectomy rates as high as 27%–30%.^4^

Contemporary management of ASUC involves admission to the hospital, excluding infectious mimics, and initiating medical therapy.^5^ The standard treatment begins with intravenous steroids and if clinical response is not achieved, typically within 5 days, salvage therapy is generally recommended to reduce the risk of requiring colectomy.^6^ Guidelines recommend either infliximab or cyclosporine as rescue therapy based on centre experience, however, anti-TNF therapy has generally replaced calcineurin inhibitors given their noninferiority and greater tolerability.^7,8^

One important limitation of the current body of literature supporting the use of anti-TNF rescue therapy in ASUC is that it is derived from studies which included exclusively or mostly anti-TNF naïve patients.^9^ As such, there is limited evidence to guide the in-hospital management of persons with ASUC who are on or have been previously exposed to biologic therapy. This is a critically important knowledge gap, given that the prevalence of biologic therapy, and consequently anti-TNF use, in UC has been steadily increasing over time,^10^ suggesting that more and more patients who are admitted to hospital with ASUC will be active biologic users or would have been previously exposed. Commonly used therapeutic options include providing additional and/or accelerated dosing of the existing anti-TNF agent, switching to another advanced therapy (such as vedolizumab, ustekinumab, or JAK inhibitors), considering cyclosporine, or potentially, considering colectomy earlier in the course of management. However, the outcomes of patients on BST who receive these regimens are poorly defined.

Given this important knowledge gap, we sought to review our current practice among persons admitted to hospital with ASUC, comparing persons on existing biologic or small molecule therapy (BST) to persons not actively using these agents.

## Methods

### Data sources

We conducted an electronic, retrospective chart review of patients admitted for possible ASUC flare from January 1, 2018 until January 1, 2022 at Mount Sinai Hospital. This is a tertiary referral centre in Toronto, Ontario with expertise in the multidisciplinary care of patients with inflammatory bowel disease. Potentially eligible admissions were identified as those with an ICD-10 code for ulcerative colitis (K51.X) listed as the most responsible or primary diagnosis.^11^

### Inclusion criteria

Identified charts were initially reviewed to confirm the diagnosis of ASUC. In order to be included, patients had to meet the Truelove and Witts definition of severe UC: reported bowel movement frequency of >6 per day with at least one of fever (≥38 °C), tachycardia, anaemia (<105 g/L) or elevated inflammatory markers [CRP ≥ 10 mg/L or ESR > 30 mm/h]. Patients were also included if they were deemed to be clinically in a severe flare based on expert judgement represented in the notes on admission even if the Truelove–Witts criteria were not strictly met. Subjects that had a diagnosis of Crohn’s disease, or had undergone a colectomy were excluded.

### Data collection

For each ASUC patient admission, we collected the following information: demographic details including age, gender, and substance use history; baseline disease characteristics including disease duration, UC phenotype if available, history of UC flare requiring admission in the last year; IBD medication use history including use of 5-ASA compounds, immunomodulators, and details regarding previous and current biologic exposure; disease severity at admission based on BM frequency, vital signs, routine blood work, and inflammatory markers.

Patients were considered to be on biologic or small molecule therapy if they had received any anti-TNF agent, anti-integrin, IL-12/23 inhibitor, or JAK-inhibitor in the 56 days prior to admission; all persons who did not meet this criterion were considered for the comparator group (non-BST). We also recorded any prior exposure to biologic therapy, recording the reasons for discontinuation if available.

For each eligible admission, we collected the dose and duration of intravenous steroids received. For each biologic or advanced therapy provided in the hospital we recorded the day of the administration and dose. We measured the time from admission until provision of biologic therapy, surgical consultation, operative management, and discharge. We also reviewed the results of laboratory testing, focussing on white blood cell counts, haemoglobin, albumin, and C-reactive protein. We recorded the incidence of any of the following complications: hospital-acquired respiratory or urinary infection, *Closteroides difficile* infection, CMV colitis, toxic megacolon, sepsis, critical care unit admission, and venous thromboembolism.

### Outcomes

Our outcomes of interest included the differences in the mean hospital length of stay (HLOS), duration of intravenous steroids, proportion of patients receiving any biologic therapy and the class of biologic given. Additional outcomes include differences in time to receipt of biologic therapy and proportion requiring escalation of biologic therapy. Among the BST group, we also tracked the proportion who received further dosing of the biologic they were using immediately prior to admission as well as new biologic agents/or advanced therapies. We also compared rates of surgical consultation, in-hospital colectomy rates, and readmission within 90 days between the BST and non-BST cohorts. As our primary objective was to offer a descriptive analysis contrasting these 2 groups, we did not assign a primary outcome and thus did not conduct a formal power calculation.

### Statistical analysis

We performed our statistical analysis using R (R Development Core Team 2021). Our sample means were compared using unpaired, 2-sided *t*-tests and the Mann–Whitney *U* test when the assumption of a normal distribution was violated. Our categorical outcomes were compared using the chi-square test of independence and Fischer’s exact test.^12^

## Results

### Study population

Between January 2018 and January 2022, we identified 717 records of admission to the hospital with a listed diagnosis of ulcerative colitis (ICD10 code K51). After excluding persons admitted to surgical services, we were left with 330 potential enrollees. Of the 330 patients, 145 were excluded for being admitted with an unrelated concern or not meeting our inclusion criteria for a severe UC flare. After completing our screening process, we were left with 185 admissions for ASUC, 109 (59%) of the identified subjects were not on BST at the time of admission, whereas 76 (41%) were on BST (Figure S1).

### Baseline characteristics

A comparison of the characteristics at the time of admission between patients on BST and patients not on BST is illustrated in Table 1. In our sample, patients on BST were slightly younger (31.5 vs 34.9 years *P* = .05) and had a slightly lower duration of disease (5.4 vs 6.2 years *P* = .43) but were significantly more likely to have had an admission for a UC flare in the last year (46.1% vs 18.4% *P* < .01). Patients on BST were less likely to use 5-ASA compounds (25% vs 57.8% *P* < .01) but more likely to be on immunomodulators (21% vs 3.7% *P* <.01). Patients on BST, compared to patients not on BST, were more likely to have had previous treatment with anti-TNF agents (36.8% vs 11.9% *P* < .01) and vedolizumab (17.1% vs 2.8% *P* < .01). Of the patients admitted on BST, 47.4% were on infliximab, 6.6% were on golimumab, 10.5% were on adalimumab, 26.3% were on vedolizumab, 7.9% were on ustekinumab and 1.3% were on tofacitinib, at the time of admission (Table 1). With regards to disease severity on admission, the 2 groups were similar with regards to the presenting vital signs, rates of rectal bleeding, bowel movement frequency and endoscopic findings (Table 1). Patients on BST had a lower mean CRP at admission (41.8 mg/L vs 67.4 mg/L *P*-value < .01).

**Table 1.** Baseline characteristics and admission severity.

Patient characteristics			
	Not on BST^a^ (*N* = 109)	On BST (*N* = 76)	*P* value
Age (years)	34.9 (SD 11.8)	31.5 (SD 11.6)	.05
Males	43 (39.5%)	41 (54.0%)	.07
Current tobacco use	5 (4.6%)	3 (4.0%)	1.0
Current alcohol use	38 (34.9%)	25 (32.9%)	.90
Disease duration (years)	6.2 (SD 7.4)	5.4 (SD 5.3)	.43
UC flare requiring admission within 1 year	20 (18.4%)	35 (46.1%)	<.01
** Baseline medication use**			
Oral steroid use	29 (26.6%)	27 (35.6%)	.19
5-ASA use	63 (57.8 %)	19 (25.0%)	<.01
Immunomodulator use (AZA/MTX)	4 (3.7%)	16 (21.0%)	<.01
Previous anti-TNF therapy	13 (11.9%)	28 (36.8%)	<.01
Previous Vedolizumab therapy	3 (2.8%)	13 (17.1%)	<.01
Previous Ustekinumab therapy	0 (0%)	1 (1.3%)	.90
Previous Tofacitinib therapy	0 (0%)	0 (0%)	*N/A*
Exposure to ≥2 biologics	4 (3.7%)	29 (38.2%)	<.01
** Admission severity**			
Bowel movements ≥ 10 per day	75 (70.7%)	66 (86.8%)	.02
Systolic blood pressure ≤ 100 mm Hg	37 (33.9%)	22 (28.9%)	.58
Heart rate ≥ 100 bpm	64 (58.7%)	39 (51.3%)	.40
Temperature ≥ 38.0°C	24 (22.0%)	15 (19.7%)	.85
CRP (mg/L)	67.4 (SD 62.4)	41.8 (SD 50.1)	<.01
Haemoglobin (g/L)	118.2 (SD 23.5)	113.3 (SD 22.6)	.16
White blood cell count (10^9^/L)	11.6 (SD 3.9)	10.9 (SD 5.2)	.32
** Mayo endoscopic score** ^b^			
Mayo 1	1 (1.2%)^c^	2 (3.9%)	0.28
Mayo 2	30 (34.5%)	15 (29.4%)	0.54
Mayo 3	56 (64.4%)	34 (66.7%)	0.78
** Current biologic in patients on BST (*N* = 76)**			
Infliximab		36 (47.4%)	
Golimumab		5 (6.6%)	
Adalimumab		8 (10.5 %)	
Vedolizumab		20 (26.3%)	
Ustekinumab		6 (7.9%)	
Tofacitinib		1 (1.3%)	

^a^BST refers to biologic or small molecule therapy.

^b^Refers to the segment with the highest Mayo endoscopic score. Of the 47 patients with missing endoscopy data, 22 were from the cohort not on BST and 25 from the cohort on BST.

^c^The percentage reported corresponds to the total number of patients that have undergone endoscopy in either cohort.

### Clinical outcomes of interest

**Figure 1.**
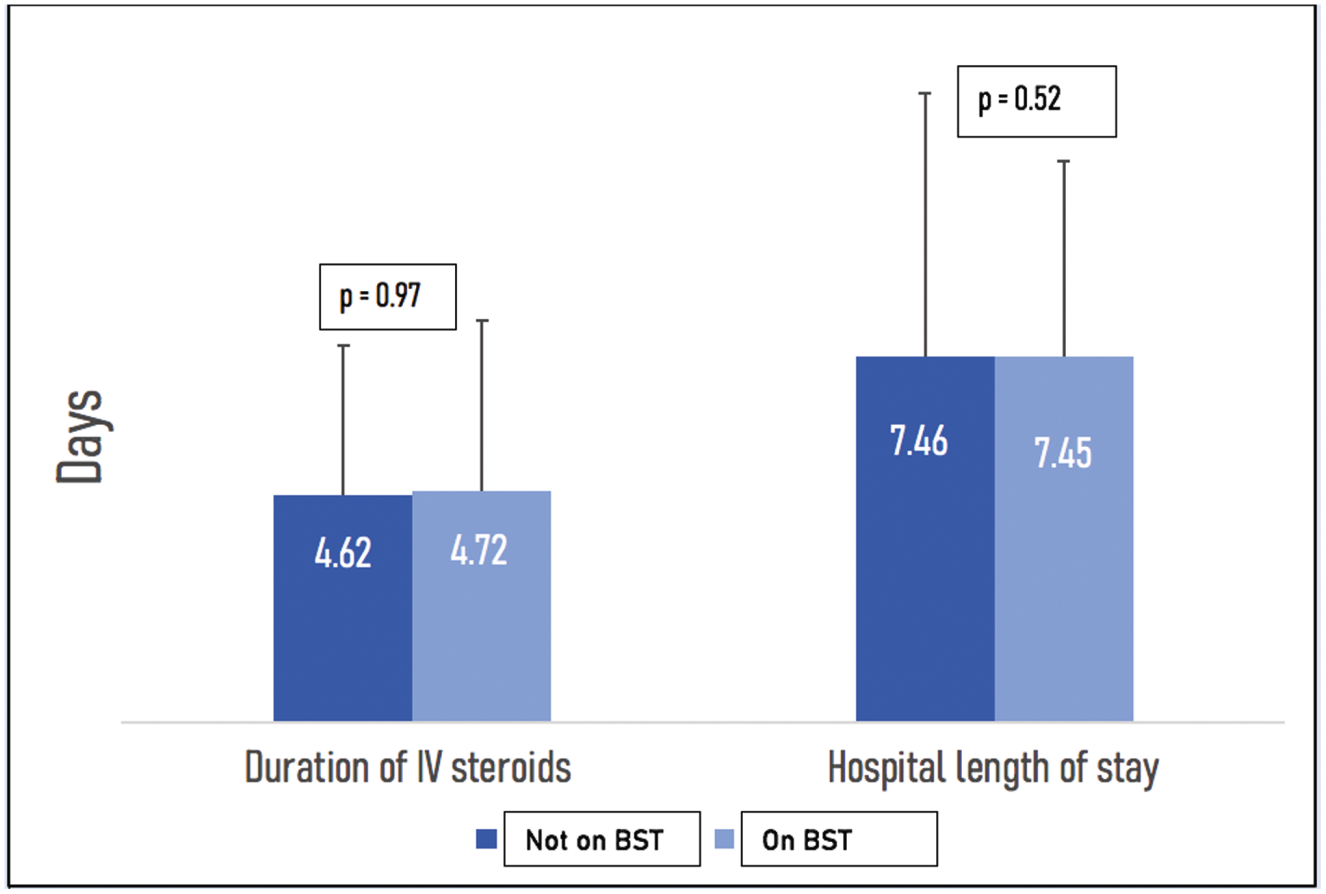
Comparison of duration of intravenous steroids and hospital length of stay in patients on biologic or small molecule therapy (BST) versus not on BST.

The primary results from our study are displayed in Table 2. We did not detect any difference in the mean hospital length of stay between persons on BST and those not on BST (7.46 days vs 7.45 days *P* = .52) (Figure 1). In-hospital colectomy rates were low in both groups with 3 patients on BST requiring colectomy compared to a single patient not on BST receiving a colectomy. There were also no differences seen in the duration, dose, and rates of reinitiation of intravenous steroids. However, patients on BST were significantly more likely to receive a surgical consultation prior to discharge (36.8% vs 8.3% *P* < .01) and were more likely to be readmitted within 90 days following hospital discharge (26.3% vs 13.8% *P* = .03).

**Table 2.** Clinical outcomes during admission for ASUC.

Outcome	Not on BST (109)	On BST (76)	*P*-value
Mean IV steroid dose (mg)	39.3 (SD 12.6)	36.5 (SD 14.5)	.10
Duration of IV steroids (days)	4.6 (SD 3.1)	4.7 (SD 3.5)	.97
Received biologic therapy	50 (45.9%)	53 (69.7%)	**<.01**
Received dose of baseline BST	*N/A*	40 (52.6%)	
Time to receipt of biologic (days)	5.4 (SD 3.4)	3.9 (SD 2.7)	**.01**
Inpatient complications^a^	3 (2.8%)	3 (4.0%)	.69
Received surgical consult	9 (8.3%)	28 (36.8%)	**<.01**
Inpatient colectomy	1 (0.9%)	3 (4.0%)	.31
Hospital length of stay (days)	7.5 (SD 5.4)	7.5 (SD 4.0)	.52
90-day readmission	15 (13.8%)	20 (26.3%)	**.03**

^a^Complications include venous thrombosis, infection, toxic megacolon, sepsis, and ICU admission.

Forty patients (52.6%) on BST received at least 1 additional dose of their biologic or small molecule therapy, whereas 13 (17.1%) received a new biologic. Of the patients on anti-TNF therapy prior to admission, 65.3% received an anti-TNF agent, 97% of whom were the same anti-TNF agent they were on prior to admission, 2.0% received ustekinumab, and 6.1% received Tofacitinib as additional therapy. Of the patients on ustekinumab therapy prior to admission, 33% received ustekinumab and 17% received tofacitinib as additional therapy (Table 3). Of the patients on vedolizumab therapy, 35% received an anti-TNF agent, and 30% received Vedolizumab as additional therapy (Table 3). Finally, the only patient on tofacitinib therapy who required additional medical therapy received tofacitinib. In comparison, of the patients not on BST that received biologic therapy, 96% received an anti-TNF agent, and 4% received vedolizumab. Only 1 patient, who was on vedolizumab therapy prior to admission, received 2 different advanced therapies.

**Table 3.** Contrasting biologic therapy received in hospital with the class of baseline biologic therapy for patients on BST.

*N* (%) (mean dose)		Biologic therapy received in the hospital					
		Infliximab	Adalimumab	Golimumab	Ustekinumab	Vedolizumab	Tofacitinib
**Baseline biologic therapy**	Infliximab *n* = 36	25 (69%) 682 mg IV	0	0	1 (3%) 260 mg SC	0	3 (8%) 10 mg BID
	Adalimumab *n* = 8	1 (17%) 500 mg IV	5 (83%) 72 mg SC	0	0	0	0
	Golimumab *n* = 5	0	0	1 (100%) 100 mg SC	0	0	0
	Ustekinumab *n* =6	0	0	0	2 (33%) 325 mg IV		1 (17%) 10 mg BID
	Vedolizumab *n* = 20	7 (35%) 460 mg IV	0	0	0	6 (30%) 300 mg IV	0
	Tofacitinib *n* =1	0	0	0	0	0	1 (100%) 10 mg BID

## Discussion

In this retrospective study of patients admitted to Mount Sinai Hospital with an acute, severe ulcerative colitis flare, we did not identify any significant differences in the cumulative dose or duration of administered intravenous corticosteroids, hospital length of stay, rates of complications, or rates of inpatient colectomy, between patients on BST compared to patients not on BST. We did find a statistically significant increase in rates of surgical consultation and hospital readmission among patients on BST.

Our results suggest that patients admitted to our centre with ASUC have similar courses in the hospital irrespective of whether they were on BST at the time of admission. The lack of differences observed in many key outcomes between these 2 groups may in part be due to methodologic shortcomings of our study. First, despite the paucity of literature regarding the true rate of many of our outcomes in this population, our sample size was likely still insufficient to detect statistically significant differences in several of our key outcomes. Additionally, although unlikely, it is possible that we may have misclassified the BST status of our patients prior to admission if their history of BST use was not accurately recorded in the medical record. This may further minimize potentially observed differences between the 2 groups. Furthermore, we were unable to ascertain colectomies which may have been performed following the index hospitalization if it was performed at another hospital.

Our findings of increased rates of surgical consultation for patients on pre-existing BST therapy, despite otherwise similar measures of severity on presentation, may indicate a high degree of concern by clinicians regarding the limited alternative medical therapies available which may increase the need to be mindful of the need for surgical rescue therapy. In steroid refractory ASUC, guidelines recommend the use of infliximab to achieve clinical remission and reduce the risk of colectomy.^8^ Given that over half of our patients on BST were already on an anti-TNF agent when they presented with ASUC, treating clinicians may espouse doubt that the provision of further anti-TNF therapy will result in clinical remission. We also noted that readmission rates were higher among persons on BST. Although it is possible that this reflects a lower rate of sustained remission in this group, the threshold for readmission may also be different for these 2 groups, such that clinicians believe there to be fewer medical options for patients already failing biologic therapy.

In patients with ASUC treated with rescue medical therapy, several strategies are emerging as alternatives or adjuncts to standard treatment. Although primarily supported by nonrandomized studies, the use of an accelerated dosing schedule of infliximab has emerged as a potential therapeutic approach.^13^ Another investigational option is the use of pharmacokinetic-driven anti-TNF dosing. Compared to the use of anti-TNF agents, there is a lack of prospective data, with no reported randomized trials, evaluating the use of non-anti-TNF agents, such as ustekinumab and vedolizumab in ASUC.^14^ In our study, we identified that 30% of patients on vedolizumab or ustekinumab at baseline, received a dose of these non-TNF biologics during their admission for ASUC. Unfortunately, our data does not allow us to determine whether this was done as part of salvage medical therapy or if these patients received these agents for a different reason such as simply receiving their biologics in accordance with their pre-admission schedule or for dose optimization.

Although our study provides further insight into the management of patients admitted with ASUC on BST, there are several additional limitations that must be taken into account. As our study was retrospective, our ability to determine key predictors such as disease severity at baseline is limited to the data that was collected as part of routine patient care and may be lacking key measures that may be indicative of severity and are also predictive of adverse ASUC-related outcomes.^15^ The data available to us was also lacking granular detail regarding remote biologic exposure, including reasons for discontinuation of a given agent, and more importantly, the dose and frequency of a biologic or small molecule therapy. This likely resulted in the inadvertent grouping of patients on BST who were simply receiving dose optimization and those receiving true rescue therapy. These are 2 different clinical scenarios, with likely a different degree of ASUC severity, that require different clinical approaches to treatment. Another important limitation, that is inherent to the retrospective design of our study, is the inability to determine the motivation behind clinical decisions, such as the reasoning behind the provision of non-anti-TNF biologics to patients with ASUC. Furthermore, as part of our chart selection process, we excluded patients who were admitted to surgical services due to having toxic megacolon or perforation on presentation, though this is a relatively uncommon presentation in ASUC in the modern era.^16^ Additionally, our study reports on outcomes from a single, tertiary health centre with a dedicated IBD unit and thus is not generalizable to other institutions. Finally, we were not able to determine whether persons discharged with ASUC from our facility were subsequently admitted to other hospitals, which may underestimate our readmission rates.

Bearing these limitations in mind, our study demonstrated that patients on BST had similar courses in the hospital when compared to those not on BST. Our findings of increased readmission rates in patients on biologic therapy, despite the similar duration of hospitalization, suggest utilizing different methods for assessing discharge readiness and importantly, ensuring prompter reassessment in the clinic following discharge, in patients on BST. ASUC remains a challenging clinical entity to manage and further prospective studies evaluating the underlying factors that contribute to readmission in patients on BST in hospital are needed.

## Supplementary data

Supplementary data are available at *Journal of the Canadian Association of Gastroenterology* online.

gwae017_suppl_Supplementary_Materials

## Data Availability

The data underlying this article cannot be shared publicly due to the privacy of individuals who participated in the study. The data will be shared on reasonable request to the corresponding author.
