# Supplementary material for: Outcomes of patients admitted with acute, severe ulcerative colitis on biologic therapy: a retrospective analysis from a tertiary referral hospital
Source: J Can Assoc Gastroenterol. 2024 May 31;7(4):306–11. doi: 10.1093/jcag/gwae017 (PMC11317625; doi:10.1093/jcag/gwae017)
Supplement: gwae017_suppl_Supplementary_Materials [file gwae017_suppl_supplementary_materials.zip › STROBE-checklist .docx]

STROBE Statement—checklist of items that should be included in reports of observational studies

|  | **Item No.** | **Recommendation** | **Page No.** | **Relevant text from manuscript** |
| --- | --- | --- | --- | --- |
| **Title and abstract** | 1 | (*a*) Indicate the study’s design with a commonly used term in the title or the abstract | Title page | “Outcomes of patients admitted with acute, severe ulcerative colitis on biologic therapy: a retrospective analysis from a tertiary referral hospital” |
|  |  | (*b*) Provide in the abstract an informative and balanced summary of what was done and what was found | 1 | “We conducted a retrospective chart review of patients admitted with ASUC to Mount Sinai Hospital (MSH) in Toronto, Ontario from January 2018 until January 2022. Included subjects were considered to be on BST if they had received a dose of these agents within 56 days prior to admission.” |
| **Introduction** |  |  |  |  |
| Background/rationale | 2 | Explain the scientific background and rationale for the investigation being reported | 3 | “As such, there is limited evidence to guide the in-hospital management of persons with ASUC who are on, or have been previously exposed to, biologic therapy.” |
| Objectives | 3 | State specific objectives, including any prespecified hypotheses | 4 | “Given this important knowledge gap, we sought to review our current practice among persons admitted to hospital with ASUC, comparing persons on existing biologic or small molecule therapy (BST) to persons not actively using these agents.” |
| **Methods** |  |  |  |  |
| Study design | 4 | Present key elements of study design early in the paper | 4 | “We conducted an electronic, retrospective chart review of patients admitted for possible ASUC flare from January 1^st^, 2018 until January 1^st^, 2022 at Mount Sinai Hospital.” |
| Setting | 5 | Describe the setting, locations, and relevant dates, including periods of recruitment, exposure, follow-up, and data collection | 4 | “We conducted an electronic, retrospective chart review of patients admitted for possible ASUC flare from January 1^st^, 2018 until January 1^st^, 2022 at Mount Sinai Hospital.” |
| Participants | 6 | (*a*) *Cohort study*—Give the eligibility criteria, and the sources and methods of selection of participants. Describe methods of follow-up  *Case-control study*—Give the eligibility criteria, and the sources and methods of case ascertainment and control selection. Give the rationale for the choice of cases and controls *Cross-sectional study*—Give the eligibility criteria, and the sources and methods of selection of  participants | 4 | “Potentially eligible admissions were identified as those with an ICD-10 code for ulcerative colitis (K51.X) listed as the most-responsible or primary diagnosis^11^. Identified charts were initially reviewed to confirm the diagnosis of ASUC. In order to be included, patients had to meet the Truelove and Witts definition of severe UC” |
|  |  | (*b*) *Cohort study*—For matched studies, give matching criteria and number of exposed and unexposed  *Case-control study*—For matched studies, give matching criteria and the number of controls per  case |  |  |
| Variables | 7 | Clearly define all outcomes, exposures, predictors, potential confounders, and effect modifiers.  Give diagnostic criteria, if applicable | 5 | “Our outcomes of interest included the differences in the mean hospital length of stay (HLOS), duration of intravenous steroids, proportion of patients receiving any biologic therapy and the class of biologic given. Additional outcomes include differences in time to receipt of biologic therapy and proportion requiring escalation of biologic therapy.” |
| Data sources/ measurement | 8* | For each variable of interest, give sources of data and details of methods of assessment (measurement). Describe comparability of assessment methods if there is more than one group | 5 | “For each ASUC patient admission, we collected the following information: demographic details including age, gender, substance use history; baseline disease characteristics including disease duration, UC phenotype if available, history of UC flare requiring admission in the last year; IBD medication use history including use of 5-ASA compounds, immunomodulators, and details regarding previous and current biologic exposure; disease severity at admission based on BM frequency, vital signs, routine blood work, and inflammatory markers.” |
| Bias | 9 | Describe any efforts to address potential sources of bias | N/A (descriptive study) |  |
| Study size | 10 | Explain how the study size was arrived at | N/A (descriptive study) |  |

Continued on next page

| Quantitative variables | 11 | Explain how quantitative variables were handled in the analyses. If applicable, describe which groupings were chosen and why | 6 |  |
| --- | --- | --- | --- | --- |
| Statistical methods | 12 | (*a*) Describe all statistical methods, including those used to control for confounding | 6 | “We performed our statistical analysis using R (R Development Core Team 2021). Our sample means were compared using unpaired, two-sided T-tests and the Mann-Whitney U test when the assumption of a normal distribution was violated. Our categorical outcomes were compared using the chi-square test of independence and Fischer’s exact test^12^.” |
|  |  | (*b*) Describe any methods used to examine subgroups and interactions | N/A no subgroups evaluated |  |
|  |  | (*c*) Explain how missing data were addressed | 6 | Patients with missing data for relevant outcomes were excluded from the study |
|  |  | (*d*) *Cohort study*—If applicable, explain how loss to follow-up was addressed  *Case-control study*—If applicable, explain how matching of cases and controls was addressed *Cross-sectional study*—If applicable, describe analytical methods taking account of sampling strategy | N/A |  |
|  |  | (*e*) Describe any sensitivity analyses | N/A |  |
| **Results** |  |  |  |  |
| Participants | 13* | (a) Report numbers of individuals at each stage of study—eg numbers potentially eligible, examined for eligibility, confirmed eligible, included in the study, completing follow-up, and analysed | 6 | “Between January 2018 to January 2022, we identified 717 records of admission to hospital with a listed diagnosis of ulcerative colitis (ICD10 code K51). After excluding persons admitted to surgical services, we were left with 330 potential enrollees. Of the 330 patients, 145 were excluded for being admitted with an unrelated concern or not meeting our inclusion criteria for a severe UC flare. After completing our screening process, we were left with 185 admissions for ASUC, 109 (59%) of identified subjects were not on BST at the time of admission, whereas 76 (41%) were on BST (figure S1).” |
|  |  | (b) Give reasons for non-participation at each stage | 6 |  |
|  |  | (c) Consider use of a flow diagram | Figure S1 (supplement) |  |
| Descriptive data | 14* | (a) Give characteristics of study participants (eg demographic, clinical, social) and information on exposures and potential confounders | 6 | “A comparison of the characteristics at the time of admission between patients on BST and patients not on BST is illustrated in Table 1.” |
|  |  | (b) Indicate number of participants with missing data for each variable of interest | N/A |  |
|  |  | (c) *Cohort study*—Summarise follow-up time (eg, average and total amount) | N/A |  |
| Outcome data | 15* | *Cohort study*—Report numbers of outcome events or summary measures over time | 7 | “The primary results from our study are displayed in Table 2.” |
|  |  | *Case-control study—*Report numbers in each exposure category, or summary measures of exposure |  |  |
|  |  | *Cross-sectional study—*Report numbers of outcome events or summary measures |  |  |
| Main results | 16 | (*a*) Give unadjusted estimates and, if applicable, confounder-adjusted estimates and their precision (eg, 95% confidence interval). Make clear which confounders were adjusted for and why they were included | 7 | “We did not detect any difference in the mean hospital length of stay between persons on BST and those not on BST (7.46 days vs 7.45 days p = 0.52). In” |
|  |  | (*b*) Report category boundaries when continuous variables were categorized |  | |
|  |  | (*c*) If relevant, consider translating estimates of relative risk into absolute risk for a meaningful time  period |  | |

Continued on next page

| Other analyses | 17 | Report other analyses done—eg analyses of subgroups and interactions, and sensitivity analyses |  |  |
| --- | --- | --- | --- | --- |
| **Discussion** |  |  |  |  |
| Key results | 18 | Summarise key results with reference to study objectives | 7 | “In this retrospective study of patients admitted to Mount Sinai Hospital with an acute, severe ulcerative colitis flare, we did not identify any significant differences in the cumulative dose or duration of administered intravenous corticosteroids, hospital length of stay, rates of complications, or rates of inpatient colectomy, between patients on BST compared to patients not on BST. We did find a statistically significant increase in rates of surgical consultation and hospital readmission among patients on BST.” |
| Limitations | 19 | Discuss limitations of the study, taking into account sources of potential bias or imprecision. Discuss both direction and magnitude of any potential bias | 9 | “Although our study provides further insight in the management of patients admitted with ASUC on BST, there are several additional limitations that must be taken into account” |
| Interpretation | 20 | Give a cautious overall interpretation of results considering objectives, limitations, multiplicity of  analyses, results from similar studies, and other relevant evidence | 9 | “Bearing these limitations in mind, our study demonstrated that patients on BST had similar courses in hospital when compared to those not on BST.” |
| Generalisability | 21 | Discuss the generalisability (external validity) of the study results | 9 | “Additionally, our study reports on outcomes from a single, tertiary health center with a dedicated IBD unit and thus not generalizable to other institutions.” |
| **Other information** | | |  |  |
| Funding | 22 | Give the source of funding and the role of the funders for the present study and, if applicable, for the original study on which the present article is based | 1 | Title page |

*Give information separately for cases and controls in case-control studies and, if applicable, for exposed and unexposed groups in cohort and cross-sectional studies.

**Note:** An Explanation and Elaboration article discusses each checklist item and gives methodological background and published examples of transparent reporting. The STROBE checklist is best used in conjunction with this article (freely available on the Web sites of PLoS Medicine at [http://www.plosmedicine.org/,](http://www.plosmedicine.org/) Annals of Internal Medicine at [http://www.annals.org/,](http://www.annals.org/) and Epidemiology at [http://www.epidem.com/).](http://www.epidem.com/)) Information on the STROBE Initiative is available at [www.strobe-statement.org.](http://www.strobe-statement.org/)
