# Supplementary figures and images for: Outcomes of patients admitted with acute, severe ulcerative colitis on biologic therapy: a retrospective analysis from a tertiary referral hospital
Source: J Can Assoc Gastroenterol. 2024 May 31;7(4):306–11. doi: 10.1093/jcag/gwae017 (PMC11317625; doi:10.1093/jcag/gwae017)

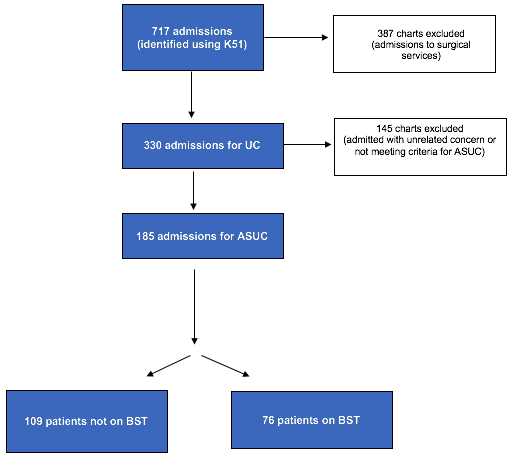

Supplement: gwae017_suppl_Supplementary_Materials [file gwae017_suppl_supplementary_materials.zip › gwae017_suppl_Supplementary.tiff]
